# Facilitators and barriers to post-discharge pain assessment and triage: a qualitative study of nurses’ and patients’ perspectives

**DOI:** 10.1186/s12913-021-07031-w

**Published:** 2021-09-28

**Authors:** Jinying Chen, Jessica G. Wijesundara, Angela Patterson, Sarah L. Cutrona, Sandra Aiello, David D. McManus, M. Diane McKee, Bo Wang, Thomas K. Houston

**Affiliations:** 1 Department of Population and Quantitative Health Sciences, University of Massachusetts Chan Medical School, 368 Plantation Street, Worcester, MA 01605 USA; 2 UMass Memorial Health Care, Worcester, MA USA; 3 Department of Medicine, University of Massachusetts Chan Medical School, Worcester, MA USA; 4 Department of Family Medicine and Community Health, University of Massachusetts Chan Medical School, Worcester, MA USA; 5 Department of Internal Medicine, Wake Forest School of Medicine, Winston-Salem, NC USA

**Keywords:** Transitional care, Symptom assessment, Pain, Cardiovascular disease, Qualitative, Natural language processing

## Abstract

**Background:**

After hospital discharge, patients can experience symptoms prompting them to seek acute medical attention. Early evaluation of patients’ post-discharge symptoms by healthcare providers may improve appropriate healthcare utilization and patient safety. Post-discharge follow-up phone calls, which are used for routine transitional care in U.S. hospitals, serve as an important channel for provider-patient communication about symptoms. This study aimed to assess the facilitators and barriers to evaluating and triaging pain symptoms in cardiovascular patients through follow-up phone calls after their discharge from a large healthcare system in Central Massachusetts. We also discuss strategies that may help address the identified barriers.

**Methods:**

Guided by the Practical, Robust, Implementation and Sustainability Model (PRISM), we completed semi-structured interviews with 7 nurses and 16 patients in 2020. Selected nurses conducted (or supervised) post-discharge follow-up calls on behalf of 5 clinical teams (2 primary care; 3 cardiology). We used thematic analysis to identify themes from interviews and mapped them to the domains of the PRISM model.

**Results:**

Participants described common facilitators and barriers related to the four domains of PRISM: Intervention (I), Recipients (R), Implementation and Sustainability Infrastructure (ISI), and External Environment (EE). Facilitators include: (1) patients being willing to receive provider follow-up (R); (2) nurses experienced in symptom assessment (R); (3) good care coordination within individual clinical teams (R); (4) electronic health record system and call templates to support follow-up calls (ISI); and (5) national and institutional policies to support post-discharge follow-up (EE). Barriers include: (1) limitations of conducting symptom assessment by provider-initiated follow-up calls (I); (2) difficulty connecting patients and providers in a timely manner (R); (3) suboptimal coordination for transitional care among primary care and cardiology providers (R); and (4) lack of emphasis on post-discharge follow-up call reimbursement among cardiology clinics (EE). Specific barriers for pain assessment include: (1) concerns with pain medication misuse (R); and (2) no standardized pain assessment and triage protocol (ISI).

**Conclusions:**

Strategies to empower patients, facilitate timely patient-provider communication, and support care coordination regarding pain evaluation and treatment may reduce the barriers and improve processes and outcomes of pain assessment and triage.

**Supplementary Information:**

The online version contains supplementary material available at 10.1186/s12913-021-07031-w.

## Background

Cardiovascular disease (CVD) affects over 121 million (48.0%) adult (≥20 year of age) Americans [1]. More than 4.8 million yearly hospitalizations occurred with CVD as the principal diagnosis [1]. Transitions from inpatient care to home are challenging for cardiovascular patients [2–4]. After hospital discharge, patients often experience symptoms that prompt them to seek acute medical attention [2–6]. These symptoms included warning symptoms related to patients’ heart conditions, and also new symptoms or exacerbations of chronic problems that could be managed in primary care [2–6]. The former may represent an appropriate, and at times unavoidable, reason for repeat visit to acute care; while the latter might be prevented by close follow-up assessment, management, and health coaching [3–6].

Symptom assessment is an integral part of evidence-based transitional care interventions [7–12]. Early evaluation of patients’ post-discharge symptoms may improve patient safety, health-related quality of life, and health service utilization [3–6, 9–12]. Follow-up phone calls are a common component of transitional care interventions and serve as an important channel for provider-patient communication about symptoms [7–10, 13]. In 2013, Medicare started reimbursing the Transitional Care Management (TCM) services, which included follow-up phone calls conducted by clinical staff [14]. These follow-up calls were used by clinical staff to schedule patients’ follow-up appointments and assess patients’ medication and health conditions. However, whether it is sufficient to use these follow-up calls as a major means for post-discharge symptom assessment remains to be investigated. The goal of this study was to assess the facilitators and barriers to evaluating and triaging general (often non-cardiac) pain symptoms in cardiovascular patients through follow-up phone calls after their discharge from a large healthcare system in Central Massachusetts. We focused on pain assessment for two reasons. First, pain (both cardiac and non-cardiac) is prevalent in cardiovascular patients after hospital discharge [15–19]. Second, prior studies showed that post-discharge general pain was associated with low health-related quality of life [19, 20], high healthcare utilization [3, 18, 21–23], and early readmission to acute care settings in cardiovascular patients [24, 25]. In our previous study, using data collected through daily automated telephone assessment of symptoms in heart failure patients [26], we found that patient-reports of post-discharge general pain were associated with increased risk of 30-day acute care use [24]. Further, the majority of patients who reported pain and were re-hospitalized were readmitted through emergency department (ED) [24]. This could involve scenarios where patients came back to the ED first due to pain, but the subsequent readmission was primarily related to the underlying heart failure symptoms (notably in the context that the ED physician may not have full context of the underlying heart failure severity and status at discharge).

Within this context, we aimed to assess facilitators and barriers of implementing a post-discharge pain assessment and triage intervention and use this information to design an implementation program (i.e., a set of implementation strategies) to support implementation of routine assessments with goal of identifying and managing non-urgent pain symptoms in the outpatient setting to prevent recurrent acute care service utilization. We conducted qualitative interviews with patients discharged after hospital admissions related to cardiac problems and with clinical teams in both primary care and cardiology clinics who frequently follow up with such patients.

## Methods

### Study design

We conducted a qualitative, formative study to identify facilitators and barriers of pain assessment and triage in cardiovascular patients. We interviewed nurses (who followed up with post-discharge patients) and patients, because effective symptom control requires actions from both healthcare providers (e.g., assessing and triaging symptoms) and patients (e.g., reporting symptoms) [10]. The study was approved by the Institutional Review Board (IRB) at University of Massachusetts Chan Medical School. We followed the Standards for Reporting Qualitative Research Checklist [27] (Additional file 1) when preparing this manuscript.

### Theoretical framework

Our study was guided by the Practical and Robust Implementation and Sustainability Model (PRISM) [28]. In addition, the Coleman Care Transition Model (CTM) [10] was used to enhance the PRISM model to design and analyze patient interviews.

PRISM is a comprehensive model for translating research into practice [28], which incorporates concepts from quality improvement [29, 30], chronic care [31], the diffusion of innovations [32] and the outcome measures from the Reach, Effectives, Adoption, Implementation and Maintenance (RE-AIM) framework [33]. The PRISM model describes four domains that influence the success of an implementation effort: (1) the program or intervention; (2) external environment; (3) implementation and sustainability infrastructure; and (4) recipients. It emphasizes perspectives and characteristics of both organizational recipients (including healthcare providers) and patients. Note that PRISM’s Intervention domain focuses on implementation programs or strategies, which sometimes are also called implementation interventions [34]. We used PRISM to (1) inform the design and analysis of both provider and patient interviews; (2) guide the development of themes and align themes identified in provider and patient interviews; and (3) explore implementation strategies to support post-discharge pain assessment.

The Coleman’s CTM model [10] emphasizes 4 pillars for care transition: (1) medication self-management; (2) patient-owned/maintained health record to facilitate cross-site information transfer; (3) follow-up with primary or specialty care; and (4) knowledge by the patient or caregiver regarding warning symptoms indicative of a worsening condition and instructions on how to respond to them. We used the CTM (focusing on pillars 3 and 4) to enhance our focus when designing and analyzing patient interviews (see details in Data Collection and Data Analysis).

### Study setting and sample

We recruited healthcare providers and patients from an academic hospital (with 991 beds), which is the largest healthcare system in Central Massachusetts and serves the majority of patients hospitalized with cardiovascular diseases in this region.

#### Clinical workflows of post-discharge follow-up

The primary care clinics affiliated with the studied hospital used the billable TCM model supported by the Center of Medicare and Medicaid Services (CMS) [8, 14]. To meet criteria for billing the TCM code, patients must be contacted within 2 business days and must be seen by healthcare providers within 7 or 14 days post-discharge [14]. These primary care providers were notified of their patients’ hospital admission and discharge by Provider Activity Reports. Nurses were then assigned to follow up with their patients and schedule a follow-up appointment. The nurses were expected to have substantive, meaningful contact with the patients, including patient education, health condition assessment, and support for treatment adherence, medication management, and accessing health services. The reimbursement for TCM services varied by payers. Payers other than Medicare may cover TCM services or similar transitional care services. The transitional care workflows in primary care clinics were driven by the TCM model. However, patients who could receive transitional care were not constrained by TCM reimbursement. The cardiology clinics used their own varying protocols to follow up with patients after hospital discharge. Patients were typically called within 2–4 days post-discharge and were scheduled to see their cardiologists within 1–4 weeks. The cardiology clinics typically followed up with patients that underwent specific cardiovascular procedures (e.g., coronary artery bypass graft, valve replacement, aortic replacement and repairs, percutaneous coronary intervention, etc.).

#### Participant recruitment

Our clinical partners assisted us in identifying eligible nurses based on job responsibilities. Specifically, we sought candidate participants who were responsible for conducting post-discharge follow-up phone calls and patient triage, or who supervised these activities. To increase the variation of our sample, we recruited participants from both primary care (including internal medicine and family medicine) and cardiology (focusing on general cardiology and cardiovascular surgery) clinics. We recruited participants via an invitation email, which included a fact sheet describing the study. We then called interested participants and obtained verbal consent prior to conducting the interviews. Healthcare providers and patients participating the interviews were provided $50 and $25 gift cards, respectively, to compensate their time.

Using information from electronic health records (EHRs), we identified adult patients (> 18 years old) who satisfied the following conditions: (1) had index hospitalization with diagnoses compatible with acute coronary syndromes or heart failure between November 2018 and November 2019; (2) had ED visits or rehospitalizations within 30 days post-discharge; and (3) reported pain between index hospitalization and readmission to acute care services. Using the service provided by University of Massachusetts Chan Medical School, we obtained an initial list of candidate patients that satisfied conditions (1) and (2) and the patients’ inpatient and outpatient notes. We then used natural language processing (NLP) to identify a subset of patients from the initial list who had diagnosis codes relevant to pain (e.g., chest pain, angina, headache) or had documentation of pain in their clinical notes. We used a knowledge-driven approach [35, 36] to adapt a general-purpose clinical NLP system cTAKES [37] to pain extraction. cTAKES extracts and maps medical terms to Unified Medical Language System concepts [37]. To adapt cTAKES to the pain domain, we compiled a list of terms related to pain using input from domain experts and converted these terms to regular expressions to match cTAKES-recognized terms. In an evaluation on 200 physician-annotated clinical notes for cardiovascular patients, the system achieved 0.85 precision and 0.99 recall for classifying whether a note mentioned pain symptoms or not [35]. We called the patients identified by NLP to verify their eligibility for the study, and recruited and obtained written informed consent for patients who were eligible and willing to participate in the interview.

Participant recruitment continued until thematic saturation was reached.

### Data collection

We conducted semi-structured interviews with both healthcare providers and patients. The interview questions were guided by PRISM and Coleman’s CTM model (see Additional file 2 for the interview guides). Table 1 shows example interview questions linked to the domains of the two models. Note that an interview question can assess more than one domain (see footnote in Table 1).

**Table 1:** Example interview questions

Model Domain	Example Interview Questions
**PRISM**	
Intervention	
Provider Perspective	How important do you think it is to do a follow-up call with patients after they are discharged from the hospital? (provider interview)
Patient Perspective	Would you like someone from the hospital to give you a follow-up call after you were discharged from the hospital? (patient interview)
Recipients	
Provider Characteristics	What sorts of challenges have you or your team experienced with this procedure (making the follow-up phone call)? (provider interview)^a^
Patient Characteristics	Was there anything you felt most challenging during the days after you were discharged from the hospital and before your recent readmission to the hospital? (patient interview)
Implementation and Sustainability Infrastructure	In addition to the follow-up phone call, are there other programs or protocols used in your practice to help the transition of patients from inpatient settings to outpatient settings? (provider interview)
**Coleman’s Care Transition Model**	
Provider follow-up	Did anyone from the hospital follow up with you about your condition within the first week after you were discharged from the hospital? (patient interview)
Symptom management	Was there any reason that you did not talk to a doctor about this change (new pain)? (patient interview)

^a^ This question can also assess barriers related to external environment and infrastructure

For provider interviews, we queried the providers’ experience with the follow-up phone calls and symptom assessment, and their opinions about the usefulness and challenges of conducting these activities. For patient interviews, we queried the patients’ experiences around the index hospitalization and readmissions, focusing on symptoms and self-management. Because pain assessment is part of symptom assessment, we adopted a two-layer (or two-stage) approach for the interviews, where we first asked questions related to general symptom assessment and then narrowed down our questions to pain symptoms and assessment. Informed by narrative research [38–40], we designed some interview questions in the style of narrative queries. For example, we asked providers: “Could you tell me about your experience with the follow-up call?” and asked patients: “Could you tell me what happened on the day that you were readmitted to the hospital (or the Emergency Department)?” Our intention was to allow the interviewees to share their experiences in an open and natural format, which would give us a richer context for identifying facilitators and barriers of symptom (or pain) assessment. Because most patient interview questions are in this style (see Additional file 2), we used the guide from CTM to enhance their focus (i.e., setting the scope of these questions within patients’ experiences with hospital follow-up and symptom management).

Interviews took place from January 2020 through May 2020. Twenty-two of the interviews were conducted by JC (with training in Health Informatics and Implementation Science). One patient interview was conducted by JW (with training in Public Health and Health Education) and JC. Four interviews were conducted in-person with healthcare providers; the other interviews were conducted via phone. On average, patient interviews lasted 23 min and provider interviews lasted 31 min.

All persons collecting or handling data were trained in human subjects’ procedures, confidentiality, and privacy protection. We used the participant’s first name only during the interview to protect the privacy of the participant. The data for participant recruitment and interviews were kept in secured databases and computers accessible only to research staff with approved access.

Audio recordings of interviews were transcribed verbatim without subject identifiers by a professional transcriptionist (approved by the IRB). The first names were replaced by a generic name holder (i.e., #name#) in the transcripts.

### Data analysis

We analyzed the interview transcripts qualitatively in an iterative process by using a deductive-inductive thematic analysis approach [41, 42]. When coding the provider interviews, JC read through the interview data and developed an initial codebook using guides from PRISM but not fully constrained by the model. JC and JW coded the first interview independently using this initial codebook, discussed the coding results, and refined the codebook. AP (with training in Psychology and Nursing) used the refined codebook to code subsequent interview transcripts. The coded transcripts were reviewed and revised twice by JW and JC, respectively. Discrepancies were discussed among AP, JW, and JC during weekly coding meetings until reaching a consensus. The codebook was revised by adding new codes or revising existing codes when necessary. After the first-round coding, JC re-examined the codes: merged similar codes, dropped less relevant codes, and identified themes. JW reviewed the new changes to the codes and codebook. Discrepancies were resolved by discussions between the two authors. Themes were developed by using the Braun & Clarke’s approach [43] and the guide from PRISM. Specifically, codes were examined to identify those codes that clearly fitted together into a theme. This process resulted in initial themes. These initial themes were then reviewed and refined according to the study’s purpose and through the lens of PRISM (i.e., whether a theme is related to a PRISM domain).

We used a similar procedure to code patient interviews and develop the themes (Additional file 3). The major difference is that both the PRISM model and the Coleman’s CTM model were used as guides for creating the initial codebook. For example, we had codes specific to the CTM domains, such as “negative (positive) experiences with follow-up calls”, “challenges with symptom management”, and “self-management challenges related to emotional tension”.

The themes identified from patient and provider interviews were mapped to the domains of the PRISM model. The larger research team discussed preliminary themes to reach consensus on final themes. All coding and analyses were conducted in QDA Miner 5.0.

## Results

### Participant characteristics

Seven nurses (three from three cardiology clinics and four from two primary care clinics) were interviewed between January and March of 2020. The nurses were all female. Two were nurse managers supervising post-discharge follow-up activities, including follow-up phone calls. The other five nurses were either conducting follow-up calls and/or triaging patients who reported issues during these calls.

Sixteen patients were interviewed between March and May of 2020. The patients’ average age was 67 (SD = 10); 9 were female (56%); 15 patients were White (94%) and 1 patient was Black (6%). Patients were interviewed 4–15 months after they were rehospitalized or returned to the ED.

### Overview of qualitative results

**Fig. 1:**
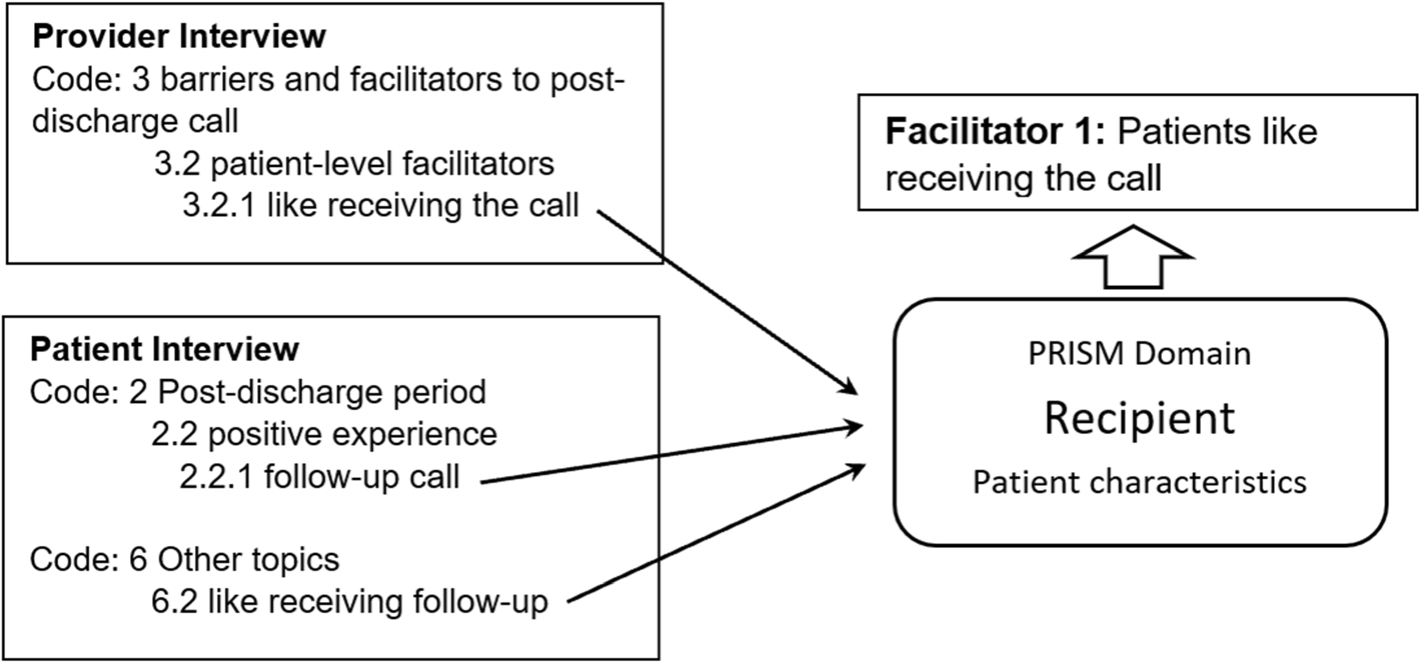
An example of using PRISM to link themes from provider and patient interviews

**Fig. 2:**
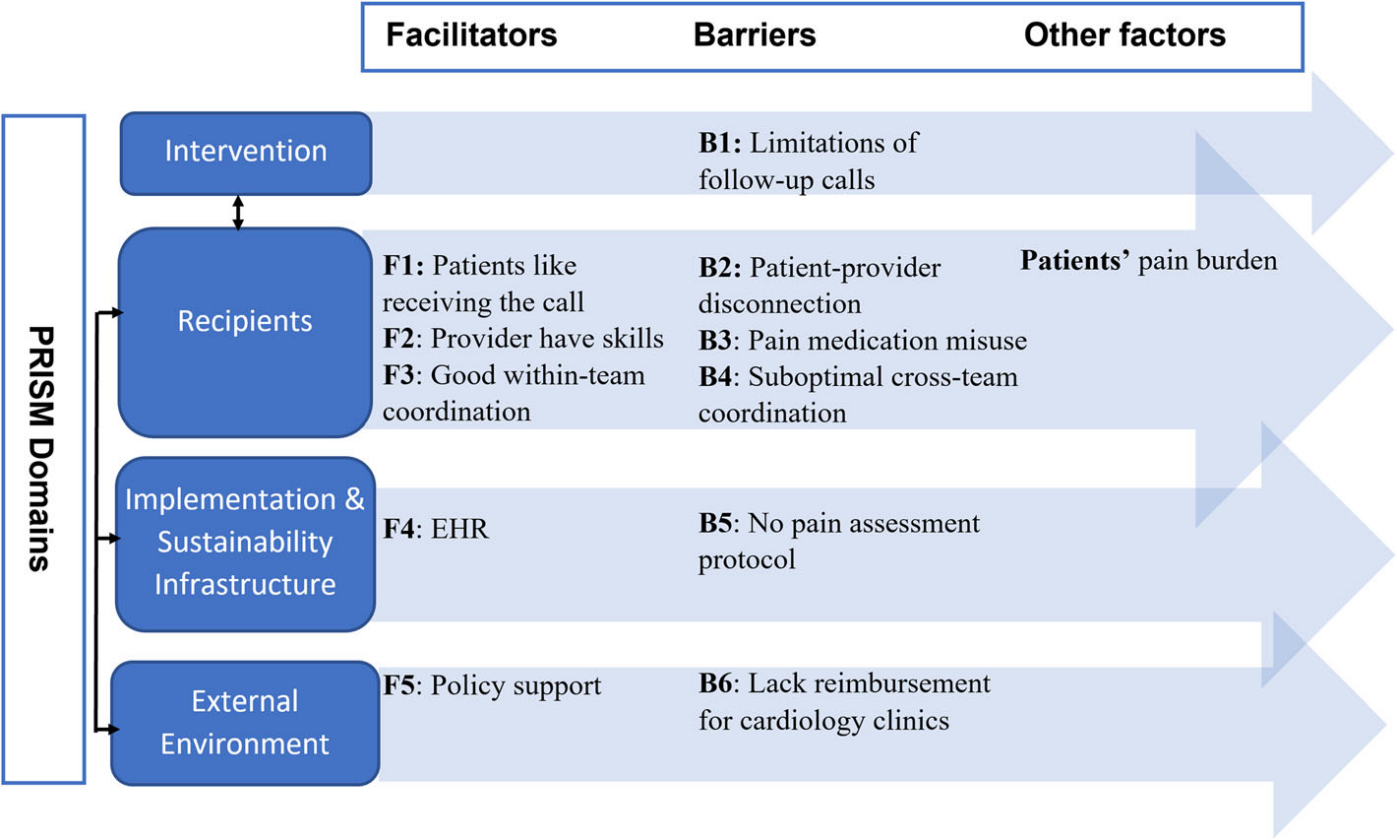
Overview of qualitative results. Full names of the themes: Facilitator 1 (F1): Patients are grateful for or willing to receive follow-up from providers. F2: Nurses have experiences and skills for assessing symptoms including pain symptoms. F3: Good coordination within clinical teams for post-discharge patient care including symptom assessment. F4: EHR system and templates for follow-up call. F5: Follow-up calls are supported by the CMS or institutional policies. Barrier 1 (B1): Symptom assessment has been part of current provider-initiated follow-up phone calls but the impact has been limited by factors such as competing priorities, coverage, and timing. B2: Providers and patients have difficulty reaching each other. B3: Concerns about pain medication misuse (pain specific). B4: Sub-optimal coordination across clinical teams for post-discharge patient care. B5: No standardized pain assessment and triage protocol for follow-up calls (pain specific). B6: Lack of emphasis on follow-up call reimbursement among cardiology clinics. Patients’ pain burden: Patients’ disease burden related to post-discharge pain symptoms

We identified themes related to: (1) patient’s disease burden related to pain and (2) barriers and facilitators to post-discharge pain assessment and triage. These themes were mapped to four PRISM domains. Figure 1 provides an example of linking themes from patient and provider interviews. Figure 2 provides an overview of the themes and their connections. These themes can form broader or higher level themes based on whether they belong to the same PRISM domain and whether they are barriers or facilitators. For example, the three themes B2 (Patient-provider disconnection), B3 (Pain medication misuse), and B4 (Suboptimal cross-team coordination) form the higher-level theme *Barriers in the PRISM’s Recipients domain*. That is, they are all barriers related to the characteristics of recipients of the implementation program.

Additionally, the connections between these themes can be partially understood through the lens of the cross-domain connections (represented by line arrows in Fig. 2) in the PRISM model, which we will discuss in greater detail in the Discussion section. We now describe the low-level themes in the next two sections.

### Patient’s disease burden related to post-discharge pain symptoms

Patient interviewees reported having various types of pain, including chest pain, surgical wound pain, and chronic pain (e.g., back and knee pain) following hospital discharge. Chest pain, both cardiac and non-cardiac, was a common symptom that drove patients to the ED. Patients also reported a heightened awareness of certain symptoms, as well as anxiety about what these symptoms might mean.

“When you've had a heart attack, everything for the next couple of weeks or so - everything becomes another heart attack. So I might've overreacted.” (Patient 1075)

“Because I just had a heart attack, so my confidence was shaken, and I was sort of being super cautious about a lotta things.” (Patient 1058)

### Facilitators and barriers of post-discharge pain assessment and triage

We identified facilitators and barriers within PRISM’s 4 domains (Fig. 2; representative quotes in Additional file 4).

#### Intervention

Our current implementation strategy to support post-discharge pain assessment and triage is to integrate a formal pain assessment and triage protocol into follow-up phone calls in the existing transitional care workflow. We aimed to use this strategy to increase feasibility and lower the incremental cost of call completion. On the other hand, our interviews revealed some limitations of this strategy.

***Barrier:*** *Symptom assessment has been part of currrent provider-initiated follow-up phone calls, but the impact has been limited by factors such as competing priorities, coverage, and timing.*

All healthcare provider interviewees recognized symptom assessment as an integral part of the follow-up phone calls. Some explicitly mentioned benefits of conducting symptom assessment during the phone call.

“I think that one of the biggest things after discharge is just touching base with the patient, number one, *seeing how they feel*.” (Provider 8).

However, the role of follow-up calls in symptom assessment was limited, partly due to competing priorities such as scheduling following-up appointments and checking medications. In addition, the follow-up calls often have a limited coverage, e.g., “only for a select group of patients” undergoing specific cardiovascular procedures (Provider 1) or due to limited resources or difficulty identifying patients discharged from other healthcare systems (Provider 3). Another potentially more problematic issue is that single, provider-initiated calls did not always capture the patient’s symptoms at the right time: “They just called to ask if I had any concerns or anything, and *I didn’t at that time*. So, I mean, being discharged from the hospital, to be honest with you, *I was really exhausted, but I didn’t have any chest pain or anything* [at that time].” (Patient 1020).

#### Recipients

We identified facilitators and barriers at the individual (patient or provider, or both) and organizational levels.

### Individual level

***Facilitator:*** *Patients were grateful for or willing to receive follow-up from providers.*

All patient interviewees received transitional care (e.g., follow-up calls, follow-up appointments, home visits, rehabiliation programs) after hospital discharge. Follow-up calls and appointments from the hospital were the most common form of transitional care received. Patients’ experiences with the follow-up calls were mostly positive. They were willing to receive the calls and valued the provider follow-ups, citing their role in rebuilding confidence and health.

“I felt like the follow-up was excellent. I don't remember the specific questions, but I do remember they did call and set up an appointment and so forth.” (Patient 1075)

“And for me there was a tremendous loss of confidence, and so anyone who wanted to reach out and check on me or help me, even reassure me to the extent that that can be done but really any communication, any opportunity to ask questions, any opportunity for somebody to give me input or advice was to me was invaluable.” (Patient 1058)

This theme was also echoed by providers.

“I really think that [the follow-up call program] was a good thing that we started here. The patients, too, 'cause we triage on the phone sometimes when we call them and stuff. They're very happy that somebody cares. Somebody's—Yeah, you can tell. They're very happy, very thankful that we're calling and checking on them.” (Provider 4)

***Facilitator:*** *Nurses have experiences and skills for assessing symptoms, including pain symptoms.*

The provider interviewees showed confidence when talking about their experiences with assessing symptoms, including pain. When patients reported symptoms, providers usually sought more information by asking questions; two respondents (Providers 1 & 8) used “tease out” when describing this process of eliciting information. They also assessed whether the patient was taking medications for pain: “We’ll try to handle it [non-cardiac pain] in the outpatient setting and try to see if he’s on, what they’ve done for medications, what’s worked for them.” (Provider 6). Some interviewees mentioned that being familiar with the patients was an advantage for the nurses when they assessed patients’ symptoms, especially pain.

“They've been in the hospital, so we know their personality. We know their history. We know what their pain was like before they came in. We have EKGs to support or to make us more concerned.” (Provider 1)

***Barrier:*** *Providers and patients have difficulty reaching each other.*

Providers identified difficulty reaching the patients as a major challenge in completing follow-up calls. They often could not reach patients during the first calls, even after trying multiple times. This issue was more disturbing when the workflow did not allow patients to call back directly.

“There's been many times that you will see in our triage notes: “Left message on machine for patient to call back to schedule a follow-up appointment.” And so that takes a lotta time. I mean, I can go a couple of days and keep calling the patient back every day and, “Left message on machine. Left message,” and then sent a message to the doctor, saying, “Would you like to send a letter to the patient?” ” (Provider 5)

“What usually happens is they call, and the patient calls back, and when the patient calls back, they don't get the nurse directly. They go into the queue, and they talk to the phone staff, and then the phone staff sends the nurse a message, and then the nurse calls back.” (Provider 8)

Patient participants described a number of situations that prevented them from reaching their providers about post-discharge symptoms prior to returning to the ED; these included delayed care, changing primary care physicians (PCPs), phone call not reaching providers directly, and patients not knowing who to call.

“Not to call 'em and wait for them to call back, right. So, very slow, didn't call back.” (Patient 1079)

“I had a primary for years, and he retired last year, so I ended up with a new primary. And when I ended up with her is when all this happened. I had the heart attack, and she since has left. So now I have a new primary who hasn't even given me a physical till July.” (Patient 1020)

***Barrier*** *(specific to pain assessment): Concerns about pain medication misuse.*

Many patients faced challenges in managing pain, especially chronic pain and recurrent chest pain.

“Well, you can't control it (old pain). It just doesn't go away.” (Patient 1007)

“Sometimes they (angina) just come, and then they go, but I wait until they really are significant before I even take a nitro.” (Patient 1040)

Some providers pointed out that pain medication misuse could be a concern or challenge when assessing pain.

“The only challenge I would anticipate, and I think - I don't think it would be major, would be for specifically patients that are [*sic*] have a history of opioid abuse. Maybe that would potentially open up a can of worms by discussing pain, because once you sort of ask the patient, and then if they're experiencing significant pain, it has to be addressed. So, I think that that would be the only obstacle.” (Provider 8)

Some patients also expressed worries about addiction to pain medications.

“No, and the only things that seem to work are narcotics, but then you're into the worry of getting addicted. Then you're into the worry of not feeling like yourself.” (Patient 1022)

### Organization level

***Facilitator:*** *Good coordination within clinical teams for post-discharge patient care, including symptom assessment.*

Many provider interviewees described teamwork within their clinics as an effective way to deliver care to post-discharge patients, especially for symptom assessment. All interviewees mentioned that they would consult attending physicians or PCPs if patients reported symptoms that they felt needed immediate attention from physicians.

“So there are times that I may not have the answers for them, but I know where to get them, and I will go directly to the primary care doctor and ask for their advice on questions that I don't feel comfortable answering.” (Provider 5)

Other instances for which care coordination was mentioned included coordination around receipt of information on patient’s hospital discharge, arrangement of post-discharge follow-up appointments, and sharing of the load for follow-up calls.

***Barrier:*** *Sub-optimal coordination across clinical teams for post-discharge patient care.*

Compared with within-team coordination, the coordination across clinical teams was less consistent and more challenging. The patients often did not expect to have a follow-up visit with their primary care providers if they had already scheduled appointments with their cadiologists.

“Sometimes they'll call back and say, ‘Well, I didn't think I needed to come to my primary care, because I had an appointment with my cardiologist.’ ” (Provider 5)

When patients were discharged from different healthcare systems, it was difficult for primary care nurses to receive the discharge notices in a timely manner.

“The notification is always the challenging part. It's like, how do we find out that a patient is being discharged? So in a hospital setting, and it's our hospital, then that's the easiest way to find out. But if they're discharged from an area hospital, or if they're discharged from a skilled nursing facility or a rehab, any kind of a rehab type of facility, we don't always get notification.” (Provider 3)

The cardiologists rarely contacted PCPs directly for patient’s hospital stay.

“On a rare occasion, I will e-mail a PCP, or my attending might call a PCP and say, ‘We had this guy,’ but it's rare.” (Provider 1)

#### Implementation and sustainability infrastructure

***Facilitator:*** *EHR system and call templates to support follow-up calls.*

All providers acknowledged the usefulness of the EHR or certain EHR features in supporting the follow-up calls, such as an auto-populated list of recently discharged patients, templates or question lists for making the follow-up calls, secure chat, and ease of access to patient medical history or other lab test results through the EHR system.

“Yes, we have questions. It used to be that we used to fill out the questions, and it didn't usually — it didn't used to populate the medications. But now it's populating the medications … ” (Provider 5)

“I'm looking at their EKGs right in front of me. I have Epic open. When I make all my phone calls, I'm in Epic.” (Provider 1)

***Barrier*** *(specific to pain assessment): no standardized pain assessment and triage protocol for follow-up calls.*

The current template for follow-up calls does not include a standardized protocol for pain assessment and triage. Nurses from different clinics used different approaches for pain assessment. Nurses from the primary care clinics have the book “Telephone Triage Protocols for Nurses” as a reference [44]. The cardiology clinics had questions in their phone call templates for specific types of pain (e.g., chest pain and surgical wound pain).

“I don't think they necessarily asked a pain question. I don't believe that's in the template, but the nurses are basically kind of doing the checking with them.” (Provider 3)

Some provider interviewees explicitly mentioned the usefulness of adding a formal pain assessment protocol during follow-up calls.

“I think that'd be great. I mean, if you have a template (for pain assessment), every time we get a call, boom, just open up the template, go right down the line of questions.” (Provider 4)

#### External environment

***Facilitator:*** *Follow-up calls were supported by CMS and institutional policies.*

Making follow-up calls to patients after their hospital discharge was a common practice across clinics in the studied hospital. These calls are required by CMS for using the billable TCM codes and they (including non-TCM calls) are supported by institutional policies.

“It's a huge priority. Actually, the doctors will sometimes send us messages that, ‘The patient's being discharged tomorrow. Make sure you do a TCM’.” (Provider 5)

“They're definitely supporting this program (follow-up call and triage). They think it's great, so I'd say it's a priority.” (Provider 6)

***Barrier:*** *Lack of emphasis on follow-up call reimbursement among cardiology clinics.*

Unlike primary care clinics, whose follow-up calls were usually billed as the TCM calls for reimbursement, cardiology clinics often relied on their own resources to sustain the follow-up calls.

“It's not reimbursable. It's part of the hospital charge.” (Provider 1)

“Yeah, unfortunately we work really closely with one of our vascular surgeons [ … ] he's tried to work with us to get payment for these phone calls and everything like that, and it's really hard to do, Medicare especially, and what we would hate to have happen is the patient get bill (from) us trying to bill something, and the insurance not cover it and the patient gets billed.” (Provider 6)

## Discussion

### Principal findings

In developing an implementation program (i.e., implementation strategies) to support post-discharge pain assessment and triage, we conducted a formative study in a large healthcare system in central Massachusetts to identify important facilitators (F1–F5 in Fig. 2) and barriers (B1–B6 in Fig. 2) to assessing and triaging post-discharge pain through telephone follow-up calls. Prior studies have shown the feasibility and benefits of using follow-up calls to assess post-surgical pain [45–49] and cancer pain [50], but provided limited detail about facilitators and barriers to the implementation process. Our study contributed to filling this knowledge gap.

We mapped the identified facilitators and barriers to four PRISM domains (Fig. 2): Intervention (I), Recipients (R), Implementation and Sustainability Infrastructure (ISI), and External Environment (EE). The connections between these factors can be considered from different perspectives, such as whether they belong to the same PRISM domain and whether they may impact the same implementation outcome(s). For example, the coverage and timing issues associated with provider-initiated calls (I/B1) and patient-provider disconnection (R/B2) can both negatively impact the reach of the intervention (i.e., post-discharge pain assessment and triage); while effective within-team coordination (R/F3) can positively impact the reach. Furthermore, the connections between these factors can be partially understood through the lens of connections between the PRISM domains (represented by line arrows in Fig. 2). For example, according to Fig. 2, factors in the Intervention (I) domain and factors in the Recipients (R) domain may impact each other. In our case, the lack of cross-team coordination (R/B4) can increase the difficulty in identifying discharged patients and, therefore, contribute to the limitations of conducting symptom assessment through follow-up phone calls (I/B1). Below we will discuss implementation strategies that may help to capitalize on facilitators and reduce barriers.

### Facilitators

Integrating quality improvement interventions into clinical settings in a cost-effective way is important but challenging [51, 52]. A major advantage of our current implementation strategy to support post-discharge pain assessment is utilizing follow-up calls in the existing transitional care workflows to reduce costs. We found that this related to most facilitators we identified. For example, the nurses conducting follow-up calls already had experience and training in triaging symptoms, including pain symptoms (R/F2). The clinics have built infrastructure (e.g., phone call templates and patient history in EHR) to support this process (ISI/F4) and have effective team coordination for transitional care (R/F3). Under these conditions, it would be less expensive to incorporate a formal pain assessment into these follow-up calls than it would be to start a new program from the scratch. These findings are compatible with those from prior studies on pain assessment in other settings [53–55]. These studies found that the experience and clinical competence of healthcare providers and collaboration between providers were important for assessing post-surgical pain in inpatient settings [53] and pain (acute or chronic) in nursing homes [55], and EHR access was crucial in triaging chest pain by phone calls in outpatient settings [54].

### Barriers, and strategies to reduce barriers

We discuss several strategies that may help reduce the barriers. Table 2 summarizes the main strategies (S1 to S8), which fall into three categories: (1) reaching patients, (2) coordinating care transition, and (3) providing support for pain care.

**Table 2:** Main strategies to address barriers

Barriers^**a**^	Strategies to Address Barriers
**Reaching patients**	
**I/B1:** Limitations of follow-up calls	**S1:** Preparing patients/consumers to be active participants **S2:** Technology-assisted symptom monitoring
**R/B2:** Patient-provider disconnection	
**Coordinating care transition**	
**R/B4**: Suboptimal cross-team coordination	**S3:** Care coordination models and new payment models
**EE/B6**: Lack reimbursement for cardiology clinics	
**Providing support for pain care**	
**R/B3**: Pain medication misuse	**S4:** Providing training on screening pain medication misuse **S5:** Providing resources for pain management **S6:** Supporting care coordination between PCPs, cardiologists, and pain specialists
**ISI/B5**: No pain assessment protocol	**S7:** Developing a pain assessment protocol **S8:** Incorporating pain screening criteria into EHR

^a^ Barriers were labeled by PRISM domain/barrier No. PRISM domains: Intervention (I), Recipients (R), Implementation and Sustainability Infrastructure (ISI), and External Environment (EE)

#### Reaching patients

Our interview data revealed that provider-initiated follow-up calls had limitations in terms of timing and coverage (I/B1). For example, these calls might not have reached patients at the time when the patients noticed new symptoms or worsening of old symptoms. The number and type of patients who received a follow-up phone call from individual clinics were also limited. The cardiology clinics typically followed up only with patients that underwent specific cardiovascular procedures. The patients reached for follow-up by the primary care clinics were mostly those patients discharged from the hospital with which the clinics were affiliated. This was due to difficulty tracking patients discharged from other healthcare systems. In addition, the primary care clinics did not have sufficient staff resources to conduct timely follow-up with all of their patients. Further, our data showed that providers and patients had difficulty reaching each other (R/B2). This was especially apparent when patients experienced symptoms post-discharge; they often did not or could not reach their providers in a timely manner. These results suggest that additional strategies are needed to improve the reach and efficiency of the intervention. We discuss two strategies below.

One potential strategy could be “preparing patients/consumers to be active participants” (S1), one of the Expert Recommendations for Implementing Change (ERIC) strategies [56]. There are several ways to implement this strategy. For example, healthcare providers can educate patients about disease-specific symptoms and how to respond during hospital discharge or post-discharge follow-up calls [7, 9–11]. They can also share educational materials and emergency contact information with patients by patient portals or printed copies.

A second strategy could be helping patients to connect with their providers by leveraging technology-assisted symptom tracking and automated feedback to patients about actions to take for symptoms (S2). Symptom tracking has the potential to engage patients in their own care to improve symptom control, patient-provider communication, and patients’ quality of life [57]. It has shown beneficial effects in patients with cancer, mental health conditions, and heart failure [58–61]. Further, it is important to understand patients’ preferences of technology use and provide multiple options for patients to report symptoms [62].

#### Coordinating care transition

We found two barriers (non-specific to pain assessment) to the implementation and maintenance of the intervention, which were both related to coordination of care transition. First, the coordination between clinical teams was suboptimal (R/B4). Care coordination is a well-known challenge in caring for cardiovascular patients, who often have complex care needs and see both PCPs and specialists [63, 64]. In this study, we found that patients often did not know or expect that they would be contacted by their primary care providers after hospital discharge. The cardiologists rarely contacted PCPs directly about patient’s hospital stay and relied on the EHR to share patient information. Our findings are consistent with prior studies that identified inadequate communication between PCPs and specialists [65–67], including deficits in information transfer at hospital discharge [66]. The suboptimal cross-team coordination also contributed to patients’ confusion about who to contact when they had symptoms. This barrier can partially be addressed by communicating with patients about possible PCP follow-ups and giving them the contact information of both their cardiologist and PCP during the hospital stay or at hospital discharge. A systemic solution may require new models for care coordination (S3). For example, researchers from the Duke-Margolis Center for Health Policy and professional societies representing primary care and cardiology proposed a conceptual framework (and new payment models) to support care coordination between primary care and cardiology providers [64]. Under this framework, different models (e.g., clinician-to-clinician consultation, PCP as primary manager, and cardiologist as primary manager) can be used to facilitate cross-team communication and collaboration, depending on the patient’s care needs.

Second, cardiology clinics often could not get reimbursement for conducting follow-up calls (EE/B6). One reason was that the cardiology clinics had their own follow-up workflows (e.g., timing of the follow-up calls and appointments), which often did not fulfill the requirements for using the TCM billing code. In principal, both cardiology and primary care providers can use the TCM code if their follow-up activities comply with the requirements [14]. However, only one provider at a time can use the TCM code for a patient during the 30 days following a single hospital discharge [14]. Therefore, even if the cardiology clinics modify their workflows to meet the TCM requirements, care coordination or new payment models [64] (S3) are needed to avoid duplicate billing.

#### Providing support for pain care

We identified two barriers specific to pain care. The first barrier, i.e., concerns with pain medication addiction (R/B3), may affect the adoption of the intervention. This is not surprising, as the challenges in treating chronic pain or pain medication misuse were well recognized [68, 69], and increased with the ongoing opioid crisis in the US and shifts in policy regarding opioids, pain, and addiction [70]. A recent study found that over 40% of surveyed primary care clinics were unwilling to take new patients currently taking opioids for chronic pain [71]. Although primary care providers were confident in assessing opioid use disorder, they had low desire to treat these patients [72]. This may be due to the lack of expertise or resources in treating these patients [72, 73]. Prior studies on preventing chronic pain after cardiac surgery [74, 75] and managing pain in older adults [55] suggested that providers’ clinical competence in assessing and triaging pain and care coordination between primary care and pain care providers are crucial for success to these programs. Considering these factors, the following strategies may help reduce this barrier. First, providing proper training to nurses that focuses on screening pain medication misuse (S4). Second, sharing pain management resources (e.g., pain management programs and specialists and evidence-based interventions for pain [76, 77]) with primary care clinics (S5). Third, promoting and supporting care coordination across cardiology, primary care, and pain management providers (S6).

The second barrier is the lack of a standardized pain assessment and triage protocol (ISI/B5), which can affect the implementation and maintenance of the intervention. To address this barrier, we are developing a protocol (Additional file 5) based on the input from provider interviews and our clinical partners (S7). The protocol recommends that clinical team members who conduct the follow-up calls follow specific steps to triage patients reporting pain. Because the screening criteria for common pain conditions have been documented in existing reference manuals [44], our protocol won’t repeat those criteria. Rather, it serves as a template to support workflow and team coordination. Another strategy to support pain assessment is to work with the hospital to incoporate screening criteria for common pain conditions in cardiovascular patients (e.g., chest pain and abdominal pain) into the EHR system to enable easy access (S8).

### Limitations and strengths

We interviewed healthcare providers and patients from a single academic hospital, which may not be generalizable to other healthcare settings. This limitation was alleviated to a certain extent because TCM calls were supported by national policy and have been widely implemented. The hospital we studied is the largest healthcare system in Central Massachusetts, serving a patient population more vulnerable (with low socioeconomic status) than that in neighboring areas. Our findings may be more informative for hospitals serving vulnerable populations.

We identified candidate patients for interview through retrospective analysis of the EHR data. Patients re-hospitalized or reused ED services 4–15 months before the interview. Therefore, our findings might be affected by patients’ recall bias.

One strength of our study is the use of theorectical models. Our study was guided by PRISM, an implementation model that has a strong theoretical base [28, 78] and has been successfully applied to implementation of health services or quality improvement programs in healthcare settings [78–81]. By using PRISM as the guide, our findings are more likely to be generalizable or comparable to similar implementation studies. Further, we enhanced PRISM by using Coleman’s CTM model to improve its relevance to the specific focus of the study (i.e., symptom assessment and transitional care). Another strength is that we used a hybrid, deductive-inductive method to analyze the interview data, which enabled us to both focus on important aspects informed by theories and use the data to inform the development of themes.

## Conclusions

By interviewing key stakeholders (providers and patients) from a large hospital in Central Massachusetts, we identified important facilitators and barriers of pain assessment and triage in post-discharge cardiovascular patients. Strategies to empower patients, facilitate timely patient-provider communication, and support care coordination between primary care, cardiology, and pain management providers may reduce the identified barriers and improve the processes and outcomes of pain assessment and triage.

## Supplementary Information

**Additional file 1.** Standards for Reporting Qualitative Research (SRQR) Checklist.

**Additional file 2.** Healthcare Provider and Patient Interview Guides.

**Additional file 3.** Data Analysis for Patient Interviews.

**Additional file 4.** Example Patient and Provider Quotes (23 Interviews).

**Additional file 5.** Pain Assessment and Triage Protocol.

## Data Availability

The qualitative data generated and/or analyzed by this study are not publicly available because they were generated in interviews with healthcare providers and patients and contained protected health information (PHI), with the expectation that participant identity would be kept confidential. De-identified transcripts of interviews may be available from the corresponding author on reasonable request.

## Abbreviations

CMS: The Center of Medicare and Medicaid Services
CTM: The Coleman Care Transition Model
ED: Emergency Department
EHR: Electronic health record
IRB: Institutional Review Board
NLP: Natural language processing
PCP: Primary Care Physician
PRISM: Practical, Robust, Implementation and Sustainability Model
TCM: Transitional Care Management
