# Supplementary material for: Facilitators and barriers to post-discharge pain assessment and triage: a qualitative study of nurses’ and patients’ perspectives
Source: BMC Health Serv Res. 2021 Sep 28;21:1021. doi: 10.1186/s12913-021-07031-w (PMC8480104; doi:10.1186/s12913-021-07031-w)
Supplement: Supplementary file 1 — Additional file 1. Standards for Reporting Qualitative Research (SRQR) Checklist. [file 12913_2021_7031_MOESM1_ESM.pdf]

Additional File 1: Standards for Reporting Qualitative Research (SRQR)* Checklist

http://www.equator-network.org/reporting-guidelines/srqr/

Notes: The page/line numbers in the following checklist refer to the positions in the revised manuscript (with the changes tracked and the "Simple Markup" view option selected)

Page/line no(s).

Title and abstract

**Title** - Concise description of the nature and topic of the study Identifying the study as qualitative or indicating the approach (e.g., ethnography, grounded theory) or data collection methods (e.g., interview, focus group) is recommended	Page 1
**Abstract** - Summary of key elements of the study using the abstract format of the intended publication; typically includes background, purpose, methods, results, and conclusions	Page 2 lines 1-25<br>Page 3 lines 1-8

Introduction

**Problem formulation** - Description and significance of the problem/phenomenon studied; review of relevant theory and empirical work; problem statement	Page 3 lines 13-26<br>Page 4 lines 1-20
**Purpose or research question** - Purpose of the study and specific objectives or questions	Page 4 lines 22-25<br>Page 5 lines 1-3

Methods

**Qualitative approach and research paradigm** - Qualitative approach (e.g., ethnography, grounded theory, case study, phenomenology, narrative research) and guiding theory if appropriate; identifying the research paradigm (e.g., postpositivist, constructivist/ interpretivist) is also recommended; rationale**	Page 5 lines 7-10<br>Page 5 lines 15-23<br>Page 6 lines 1-15
**Researcher characteristics and reflexivity** - Researchers' characteristics that may influence the research, including personal attributes, qualifications/experience, relationship with participants, assumptions, and/or presuppositions; potential or actual interaction between researchers' characteristics and the research questions, approach, methods, results, and/or transferability	Page 10 lines 11-13<br>Page 11 lines 7-8<br>Additional File 3
**Context** - Setting/site and salient contextual factors; rationale**	Page 6 lines 17-25<br>Page 7 lines 1-14
**Sampling strategy** - How and why research participants, documents, or events were selected; criteria for deciding when no further sampling was necessary (e.g., sampling saturation); rationale**	Page 7 lines 17-25<br>Page 8 lines 1-21
**Ethical issues pertaining to human subjects** - Documentation of approval by an appropriate ethics review board and participant consent, or explanation for lack thereof; other confidentiality and data security issues	Page 5 lines 11-12<br>Page 10 lines 18-25

**Data collection methods** - Types of data collected; details of data collection procedures including (as appropriate) start and stop dates of data collection and analysis, iterative process, triangulation of sources/methods, and modification of procedures in response to evolving study findings; rationale**	Page 8 line 24-16<br>Page 10 lines 11-16
**Data collection instruments and technologies** - Description of instruments (e.g., interview guides, questionnaires) and devices (e.g., audio recorders) used for data collection; if/how the instrument(s) changed over the course of the study	Page 9 Table 1<br>Page 9 lines 7-13<br>Page 10 lines 1-9, 23-25
**Units of study** - Number and relevant characteristics of participants, documents, or events included in the study; level of participation (could be reported in results)	Page 12 lines 6-15<br>Page 14 lines 16-21
**Data processing** - Methods for processing data prior to and during analysis, including transcription, data entry, data management and security, verification of data integrity, data coding, and anonymization/de-identification of excerpts	Page 10 lines 18-25<br>Page 11 lines 2-14, 21-25<br>Additional File 3
**Data analysis** - Process by which inferences, themes, etc., were identified and developed, including the researchers involved in data analysis; usually references a specific paradigm or approach; rationale**	Page 11 lines 20-25<br>Page 12 lines 1-2, 10-12<br>Additional File 3
**Techniques to enhance trustworthiness** - Techniques to enhance trustworthiness and credibility of data analysis (e.g., member checking, audit trail, triangulation); rationale**	Page 11 lines 14-19<br>Page 12 lines 1-3

Results/findings

**Synthesis and interpretation** - Main findings (e.g., interpretations, inferences, and themes); might include development of a theory or model, or integration with prior research or theory	Page 13 lines 1-10<br>Page 13, Figure 2<br>Pages 14-22<br>Page 23 lines 1-5
**Links to empirical data** - Evidence (e.g., quotes, field notes, text excerpts, photographs) to substantiate analytic findings	Pages 14-22<br>Page 23 lines 1-5

Discussion

**Integration with prior work, implications, transferability, and contribution(s) to the field -** Short summary of main findings; explanation of how findings and conclusions connect to, support, elaborate on, or challenge conclusions of earlier scholarship; discussion of scope of application/generalizability; identification of unique contribution(s) to scholarship in a discipline or field	Page 23 lines 7-25<br>Pages 24-28<br>Page 29 lines 1-4
**Limitations** - Trustworthiness and limitations of findings	Page 29 lines 6-24<br>Page 30 lines 1-2

Other

**Conflicts of interest** - Potential sources of influence or perceived influence on study conduct and conclusions; how these were managed	Page 31 lines 21-25<br>Page 32 lines 1-2
**Funding** - Sources of funding and other support; role of funders in data collection, interpretation, and reporting	Page 32 lines 4-8

*The authors of the SRQR created the SRQR by searching the literature to identify guidelines, reporting standards, and critical appraisal criteria for qualitative research; reviewing the reference lists of retrieved sources; and contacting experts to gain feedback. The SRQR aims to improve the transparency of all aspects of qualitative research by providing clear standards for reporting qualitative research.

**The rationale should briefly discuss the justification for choosing that theory, approach, method, or technique rather than other options available, the assumptions and limitations implicit in those choices, and how those choices influence study conclusions and transferability. As appropriate, the rationale for several items might be discussed together.

Reference:

O'Brien BC, Harris IB, Beckman TJ, Reed DA, Cook DA. **Standards for reporting qualitative research: a synthesis of recommendations.** *Academic Medicine*, Vol. 89, No. 9 / Sept 2014
DOI: 10.1097/ACM.0000000000000388
