# Supplementary material for: Facilitators and barriers to post-discharge pain assessment and triage: a qualitative study of nurses’ and patients’ perspectives
Source: BMC Health Serv Res. 2021 Sep 28;21:1021. doi: 10.1186/s12913-021-07031-w (PMC8480104; doi:10.1186/s12913-021-07031-w)
Supplement: Supplementary file 2 — Additional file 2. Healthcare Provider and Patient Interview Guides. [file 12913_2021_7031_MOESM2_ESM.pdf]

Additional File 2: Healthcare Provider and Patient Interview Guides

Healthcare Provider Interview Guide

Prompts are included as bullets for each primary question.

Introductions and Ground-rules (3 minutes)

[Discuss goals of this research and interview procedures]. Are you ready to talk now? If so, let's start.

Experience with Post-discharge Follow-up and Care Transition (10 minutes)

I understand that *[health care system]* implemented a post-discharge follow-up call procedure. I'd like to hear your experience with this procedure.

1. How important do you think it is to do a follow-up call with patients after they are discharged from the hospital?
    - Is this related to reducing readmissions?

2. Could you tell me about your experience with the follow-up call?

    [Prompts for if they have experience]:

    - Can you tell me about a patient who seemed to benefit from a call? In what ways do you think the follow-up call was helpful?
    - Tell me about a patient for whom it didn't seem to make a difference.
    - Do you have patients who reported problems that you helped out or could not solve? Tell me more.
    - On average, how much time did you spend on such a phone call?
    - Has there been a phone call that you've spent more time on? What is the maximum time you think available for making such a phone call? Could you give me a specific example?

    [Prompts for if they do not have experience]:

    - Would you like your practice to be involved in this procedure in the future?
    - Could you think of examples that your clinic may benefit from this procedure?
    - Could you tell me any challenges you anticipate?

3. *[If interviewee has experience with follow-up calls]:* What sorts of challenges have you or your team experienced with this procedure?

   [Prompts if "patients did not pick up the call"]:
   - Do you schedule a phone call with patients in advance?
   - Have you tried to follow up with the patient in a different day?
   - How many times would you try?

4. In addition to the follow-up phone call, are there other programs or protocols used in your practice to help transition patients from inpatient settings to outpatient settings?
   - Could you give me some examples?

5. How much of a priority is it in your clinical team to work with the hospital and patients to reduce avoidable readmissions? Has it changed over time?

6. Are there other initiatives (rather than transition care and reducing readmission) that are given higher priorities?
   - Could you give me some examples?

Feedback and Opinions on Post-discharge Pain Assessment (10 minutes)

1. What are your thoughts or experiences with post-discharge pain management?
   - What would you do if, during a follow-up call, you heard that the patient had pain?
   - How does your clinic manage patients who reported pain after they were discharged and before they had a follow-up visit in the clinic?
   - What sorts of things might your clinic do if patients reported new cardiac or chest pain after discharge? And then how about old chest pain?
   - What sorts of things might your clinic do if patients reported new non-cardiac pain? And then how about old non-cardiac pain?

2. What would you think of the impact of adding a brief pain assessment to standard follow-up calls?
   - How would you envision reports of pain be best triaged to a doctor?

Wrap-up (1 min)

Thank you so much for your time and for talking about your experiences. We will be conducting similar interviews in the coming weeks. We will use the results of this discussion to improve our study to help patients better manage their pain after leaving the hospital. Do you have any other comments or questions before we finish?

Patient Interview Guide

Prompts are included as bullets for each primary question.

Introductions and Ground-rules (3 minutes)

[*Discuss goals of this research and interview procedures].* Are you ready to talk now? If so, let's start.

Causes for Recent Readmission to Hospital or Emergency Department (5 minutes)

Our records showed that you had two hospitalizations recently, one on [date]_________ and the second one on [date]__________. First, I'd like to talk about your second hospitalization.

1. Could you tell me what happened on the day that you were readmitted to the hospital (or the Emergency Department)? Are there any severe symptoms that caused you to seek urgent care?
    - Did you notice any symptoms like shortness of breath, swelling in your legs, or pain that made you seek urgent care?

[If the patient mentioned pain as a symptom, then ask Q2].

2. Can you tell me what the pain was like (could you please describe the pain)?
    - How severe was it?
    - Where was the pain located?
    - Was there a change in pain when you arrived at the hospital?
    - On scale of 1-10, 10 being unbearable, how was the pain at the time the event happened?

Post-discharge Experiences (Pain or General Issues) (15 minutes)

[If pain was mentioned as a cause of the recent readmission in the section above, then ask Q1-Q12; otherwise, start at Q10].

1. Was the pain that drove you back to the hospital a completely new pain?

[If "No" (old/chronic pain) to Q1, then ask Q2-Q5; otherwise, ask Q6-Q8].

<u>Old/Chronic Pain Questions (Q2-Q5)</u>

2. Could you tell me more about your experience in managing the pain?
    - Did you take any pain medicine?

3. Have you noticed any sign that the pain became worse in the days before the date you sought urgent care?

    [If "Yes" to Q3, then ask Q4].

4. Have you talked with a doctor about the change in your pain?

    [If "No" to Q4, then ask Q5].

5. Was there any reason that you did not talk to a doctor about this change?

<u>New Pain Questions (Q6-Q8)</u>

6. Did the new pain occur a few days earlier before the date you sought urgent care?

    [If "Yes" to Q6, then ask Q7].

7. Have you talked with any doctor or nurse about this new pain?

    [If "No" to Q7, then ask Q8].

8. Was there any reason that you did not talk to a doctor about this change?

<u>All Types of Pain Question (Q9)</u>

9. Are there any other forms of pain that you came to the hospital with and got checked out that we did not discuss already?

General Questions about Post-discharge Experiences (Q10-Q12)

10. Was there anything you felt most challenging during the days after you were discharged from the hospital and before your recent readmission to the hospital (or emergency service)?

11. Did anyone from the hospital follow-up with you about your condition within the first week after you were discharged from the hospital?
    - Could you tell me your experience with that follow-up call (or visit)?
    - Did you discuss any issues about your symptoms, medication, or follow-up appointment with the nurse (or doctor)?

12. Would you have liked someone from the hospital to give you a follow-up call after you were discharged from the hospital?

General Issues with Index Hospitalization (5 minutes)

1. Could you tell me your experience with discharge from your last hospitalization? I mean the hospitalization before you were hospitalized (used emergency service) this time.
    - Is there anything that you felt relevant to your recent readmission to the hospital (or emergency department)?

Wrap-Up (1 min)

Thank you so much for your time and the discussion of these issues. We will be conducting similar interviews in the coming weeks. We will use the results of this discussion to improve our study to help patients better manage their pain after leaving hospital. Do you have any other comments or questions before we finish?
