# Supplementary material for: Facilitators and barriers to post-discharge pain assessment and triage: a qualitative study of nurses’ and patients’ perspectives
Source: BMC Health Serv Res. 2021 Sep 28;21:1021. doi: 10.1186/s12913-021-07031-w (PMC8480104; doi:10.1186/s12913-021-07031-w)
Supplement: Supplementary file 3 — Additional file 3. Data Analysis for Patient Interviews. [file 12913_2021_7031_MOESM3_ESM.pdf]

Additional File 3: Data Analysis for Patient Interviews

We analyzed the patient interviews qualitatively in an iterative process by using a deductive-inductive thematic analysis approach. JC (with training in Health Informatics and Implementation Science) read through the patient interview data and developed the initial codebook using guidance from the PRISM model and Coleman's CTM model. JW (with training in Public Health and Health Education) provided feedback for improving the codebook, including clarification of code definitions. JC and JW coded the first two interviews independently using this codebook, discussed the coding results, and further refined the codebook.

AP (with training in Psychology and Nursing), JW, and AL (with training in Health Science) used the refined codebook to code the remaining patient interview transcripts. The coded transcripts were reviewed and revised by JW and JC, with JC reviewing JW's coded transcripts and JW reviewing AP's and AL's coded transcripts. Discrepancies or difficult cases were discussed among AP, JW, and JC during weekly coding meetings until reaching a consensus.[1] The codebook was revised by adding new codes or revising existing codes when necessary.

JC then re-examined the codes using the lens of the PRISM: merged similar codes, dropped less relevant codes, and identified themes. JW reviewed the new changes to the codes and codebook. Discrepancies were resolved by discussions between the two authors. Themes were developed by using the Braun & Clarke's approach and the guide from PRISM. Specifically, codes were examined to identify those codes that clearly fitted together into a theme. This process resulted in initial themes. These initial themes were then reviewed and refined according to the study's purpose and through the lens of PRISM (i.e., whether a theme is related to a PRISM domain).

[1] AL coded three transcripts and attended one coding meeting before leaving the study.
