# Supplementary material for: Facilitators and barriers to post-discharge pain assessment and triage: a qualitative study of nurses’ and patients’ perspectives
Source: BMC Health Serv Res. 2021 Sep 28;21:1021. doi: 10.1186/s12913-021-07031-w (PMC8480104; doi:10.1186/s12913-021-07031-w)
Supplement: Supplementary file 4 — Additional file 4. Example Patient and Provider Quotes (23 Interviews). [file 12913_2021_7031_MOESM4_ESM.pdf]

Additional File 4: Example Patient and Provider Quotes (23 Interviews)

PRISM Domain	Barriers (B#) and Facilitators (F#)	Representative Quotes
Intervention	Symptom assessment has been part of current provider-initiated follow-up phone calls but the impact has been limited by factors such as competing priorities, coverage, and timing (B1)	Competing priorities:<br>“Well, we're getting them in so that they get re-checked here, so I would say that's a benefit for them to make sure that they're getting well.” (Provider 4)<br><br>“Sometimes people go home, and they’re still not feeling right. But, really importantly, a lotta times their medication could change in the hospital, and patients are confused and/or don’t realize what the medications are actually for. So going over their med list and talking to them about it.” (Provider 5)<br><br>Limited coverage:<br>“The follow-up call is a major component, but it's only for a select group of patients. It's not all patients. It's only the patients getting stent.” (Provider 1)<br><br>“It's only those admitted to the hospitalist program at Memorial campus. It's very much of a niche. It's a smaller population, but it's the one we probably do the best with in terms of taking care of our patients.” (Provider 3)<br><br>Timing issue:<br>“They just called to ask if I had any concerns or anything, and I didn't at that time. So, I mean, being discharged from the hospital, to be honest with you, I was really exhausted, but I didn't have any chest pain or anything [at that time].” (Patient 1020)
Recipient	Patients are grateful for or willing to receive follow-up from providers (F1)	“I felt like the follow-up was excellent. I don't remember the specific questions, but I do remember they did call and set up an appointment and so forth.” (Patient 1075)<br><br>“Were you satisfied with what they told you (during the follow-up phone calls)?” (Interviewer)<br>“Absolutely. I think UMass and everyone that works there is wonderful.” (Patient 1040)

		"I think so [would like to receive a follow-up call]. I think absolutely. I think one of the things that affects you the most is it's a big change in your life. I mean, it really can knock you on your butt, and I think any follow-up on that is just kinda reassuring to kinda feel like someone has your back and is there to answer any questions." (Patient 1054)<br><br>"And for me there was a tremendous loss of confidence, and so anyone who wanted to reach out and check on me or help me, even reassure me to the extent that that can be done but really any communication, any opportunity to ask questions, any opportunity for somebody to give me input or advice was to me was invaluable." (Patient 1058)<br><br>"I really think that [the follow-up call program] was a good thing that we started here. The patients, too, 'cause we triage on the phone sometimes when we call them and stuff. They're very happy that somebody cares. Somebody's — Yeah, you can tell. They're very happy, very thankful that we're calling and checking on them." (Provider 4)
	Nurses have experiences and skills for assessing symptoms, including pain symptoms (F2)	<u>Experienced in assessing symptoms:</u><br><br>"Sometimes they have problems with their radial artery, where they had the access for their cardiac cath., and I'll kinda tease out if it's a problem or not, maybe reassurance that this is all normal. […] Other times with the cardiac symptoms or if they're having chest pain, I can maybe help tease out, 'Is this chest pain cardiac, or is it not cardiac, or are they taking the medicines?' " (Provider 1)<br><br>"I'm not gonna say, "OK, well, because you can't – because you're having trouble breathing, are you having trouble swallowing?" I wait to see what they say to me. But if it's urgent, and I think they're gonna have enough reaction, I'm gonna say, "Can you swallow? Are you OK?" " (Provider 4)<br><br>"We'll try to handle it [non-cardiac pain] in the outpatient setting and try to see if he's on, what they've done for medications, what's worked for

		them. Why are they having pain? Is it a chronic condition, or is it something that they need to be evaluated for?" (Provider 6)<br><br><u>Familiar with patient's history:</u><br><br>"They've been in the hospital, so we know their personality. We know their history. We know what their pain was like before they came in. We have EKGs to support or to make us more concerned." (Provider 1)<br><br>"The thing about the nurses is they each cover specific providers, so they get to know the patients. So some nurses know that one person may say their pain is a five, and they know the patient, and they know if their pain is a five, they're probably in significant pain. Or they may also know if their pain is blabity-blah, then they're probably OK. So they have a good knowledge." (Provider 8)
	Providers and patients have difficulty reaching each other (B2)	<u>Difficult to reach patients:</u><br>"There's been many times that you will see in our triage notes: "Left message on machine for patient to call back to schedule a follow-up appointment." And so that takes a lotta time. I mean, I can go a couple of days and keep calling the patient back every day and, "Left message on machine. Left message," and then sent a message to the doctor, saying, "Would you like to send a letter to the patient?" " (Provider 5)<br><br>"One of the biggest problems we have is or it's not maybe a problem but an obstacle is most a lot of patients don't necessarily answer their phone." (Provider 8)<br><br>"What usually happens is they call, and the patient calls back, and when the patient calls back, they don't get the nurse directly. They go into the queue, and they talk to the phone staff, and then the phone staff sends the nurse a message, and then the nurse calls back." (Provider 8)<br><br>"Here, we get a call from the call center that we gotta call the patient back to see. They may need an appointment. We call the patient back.

		They don't answer. Then they call back. They get the call center." (Provider 4)<br><br><u>Difficult to reach providers:</u><br>"It took me three days to get a hold of the doctor, 'cause he was on a convention, and the practitioner didn't call me back." (Patient 1085)<br><br>"Not to call 'em and wait for them to call back, right. So, very slow, didn't call back." (Patient 1079)<br><br>"I had a primary for years, and he retired last year, so I ended up with a new primary. And when I ended up with her is when all this happened. I had the heart attack, and she since has left. So now I have a new primary who hasn't even given me a physical till July." (Patient 1020)<br><br>"I've never been given the information on who to call or what number." (Patient 1093)
	Concerns about pain medication misuse (B3, pain specific)	<u>Challenges in managing pain:</u><br><br>"Well, you can't control it (old pain). It just doesn't go away." (Patient 1007)<br><br>"I still get the burning to this day (since hospital discharge), so I don't know what the burning is. Is it indigestion? I don't know. Is it nerve-ending pain? I don't know, and I know what nerve-ending pain _____, too, so I don't know what the burning is. I don't think anyone can really answer that. So, I don't know if it is cardiogastric [ph] or if it's more a strain in my muscles or something. I have no idea." (Patient 1020)<br><br>"I cannot take any sort of morphine or any of those drugs, and I have a few allergies, so we just have to look at those allergies" (Patient 1044)<br><br>"Sometimes they (angina) just come, and then they go, but I wait until they really are significant before I even take a nitro." (Patient 1040)<br><br><u>Concerns with pain medication misuse:</u>

		“'Cause a lotta people are narcotic-seeking, and if we prescribe things that the PCP doesn't know about, then the PCP prescribes something. Then we can get the patient in to another negative spiral that can put them in a bad situation.” (Provider 1)<br><br>“The only challenge I would anticipate, and I think - I don't think it would be major, would be for specifically patients that are [*sic*] have a history of opioid abuse. Maybe that would potentially open up a can of worms by discussing pain, because once you sort of ask the patient, and then if they're experiencing significant pain, it has to be addressed. So, I think that that would be the only obstacle.” (Provider 8)<br><br>“No, and the only things that seem to work are narcotics, but then you're into the worry of getting addicted. Then you're into the worry of not feeling like yourself.” (Patient 1022)
	Good coordination within clinical teams for post-discharge patient care, including symptom assessment (F3)	“Then I often will speak to the attending. If there's something I'm really concerned about. I'll have to track down the attending.” (Provider 1)<br><br>“So there are times that I may not have the answers for them, but I know where to get them, and I will go directly to the primary care doctor and ask for their advice on questions that I don't feel comfortable answering.” (Provider 5)
	Sub-optimal coordination across clinical teams for post-discharge patient care (B4)	“Sometimes they'll call back and say, ‘Well, I didn't think I needed to come to my primary care, because I had an appointment with my cardiologist.’ ” (Provider 5)<br><br>“The notification is always the challenging part. It's like, how do we find out that a patient is being discharged? So in a hospital setting, and it's our hospital, then that's the easiest way to find out. But if they're discharged from an area hospital, or if they're discharged from a skilled nursing facility or a rehab, any kind of a rehab type of facility, we don't always get notification.” (Provider 3)

		"On a rare occasion, I will e-mail a PCP, or my attending might call a PCP and say, 'We had this guy,' but it's rare." (Provider 1)
Implementation and Sustainability Infrastructure	EHR system and templates to support follow-up calls (F4)	"Now there is actually a report that you can run for – to identify patients who've been discharged." (Provider 3)<br><br>"Yes, we have questions. It used to be that we used to fill out the questions, and it didn't usually — it didn't used to populate the medications. But now it's populating the medications…" (Provider 5)<br><br>"I'm looking at their EKGs right in front of me. I have Epic open. When I make all my phone calls, I'm in Epic." (Provider 1)
	No standardized pain assessment and triage protocol for follow-up calls (B5, pain specific)	"I don't think they necessarily asked a pain question. I don't believe that's in the template, but the nurses are basically kind of doing the checking with them." (Provider 3)<br><br>"When I talked with the nurse from [redacted], she showed me that in their EHR system they have different templates for assessing different types of pain. … I wonder if you notice any such templates?" (Interviewer)<br>"No, it just all goes from just kind of running through your head and going through the assessment with them." (Provider 7)<br><br>"I think we should develop that [pain assessment protocol]. I think it's something that I think a primary care office should be able to help (Triage)." (Provider 1)<br><br>"I think that'd be great. I mean, if you have a template (for pain assessment), every time we get a call, boom, just open up the template, go right down the line of questions." (Provider 4)
External Environment	Follow-up calls are supported by the CMS and institutional policies (F5)	"We know when they get discharged from the ER or from the hospital. We call it a TCM call. We have to check on them. […] I think that is the Number 1 thing that we've started here in this hospital." (Provider 4)<br><br>"It's a huge priority. Actually, the doctors will sometimes send us messages that, 'The patient's being discharged tomorrow. Make sure you do a TCM'." (Provider 5)

		"They're definitely supporting this program (follow-up call and triage). They think it's great, so I'd say it's a priority." (Provider 6)
	Lack of emphasis on follow-up call reimbursement among cardiology clinics (B6)	"It's not reimbursable. It's part of the hospital charge." (Provider 1)<br><br>"Yeah, unfortunately we work really closely with one of our vascular surgeons […] he's tried to work with us to get payment for these phone calls and everything like that, and it's really hard to do, Medicare especially, and what we would hate to have happen is the patient get bill (from) us trying to bill something, and the insurance not cover it and the patient gets billed." (Provider 6)
