# Supplementary material for: Facilitators and barriers to post-discharge pain assessment and triage: a qualitative study of nurses’ and patients’ perspectives
Source: BMC Health Serv Res. 2021 Sep 28;21:1021. doi: 10.1186/s12913-021-07031-w (PMC8480104; doi:10.1186/s12913-021-07031-w)
Supplement: Supplementary file 5 — Additional file 5. Pain Assessment and Triage Protocol. [file 12913_2021_7031_MOESM5_ESM.pdf]

Additional File 5: Pain Assessment and Triage Protocol

We developed a pain assessment and triage protocol based on input from provider interviews and our clinical partners. The protocol recommends that the clinical team members who conduct the follow-up calls follow specific steps to triage patients reporting pain. We considered the following factors when designing this protocol.

First, there have been screening criteria documented in detail in existing reference manuals for common pain conditions (e.g., chest pain and back pain).[1] We therefore did not intend to replace or repeat these criteria.

Second, the implementation of these screening criteria depends on the local context (e.g., clinics, patient populations, and characteristics of individual patients) and there is no generally agreed-upon gold-standard. We therefore designed the pain assessment and triage protocol as a template that allows the flexibility to adapt to the local context to support workflow and team coordination.

Third, the primary care clinics assess various types of pain, while the cardiology clinics focus on cardiac pain (or chest pain that needs further evaluation to rule out a cardiac etiology) and pain related to heart procedures. We therefore designed separate templates for primary care and cardiology clinics.

Pain Assessment and Triage Protocol

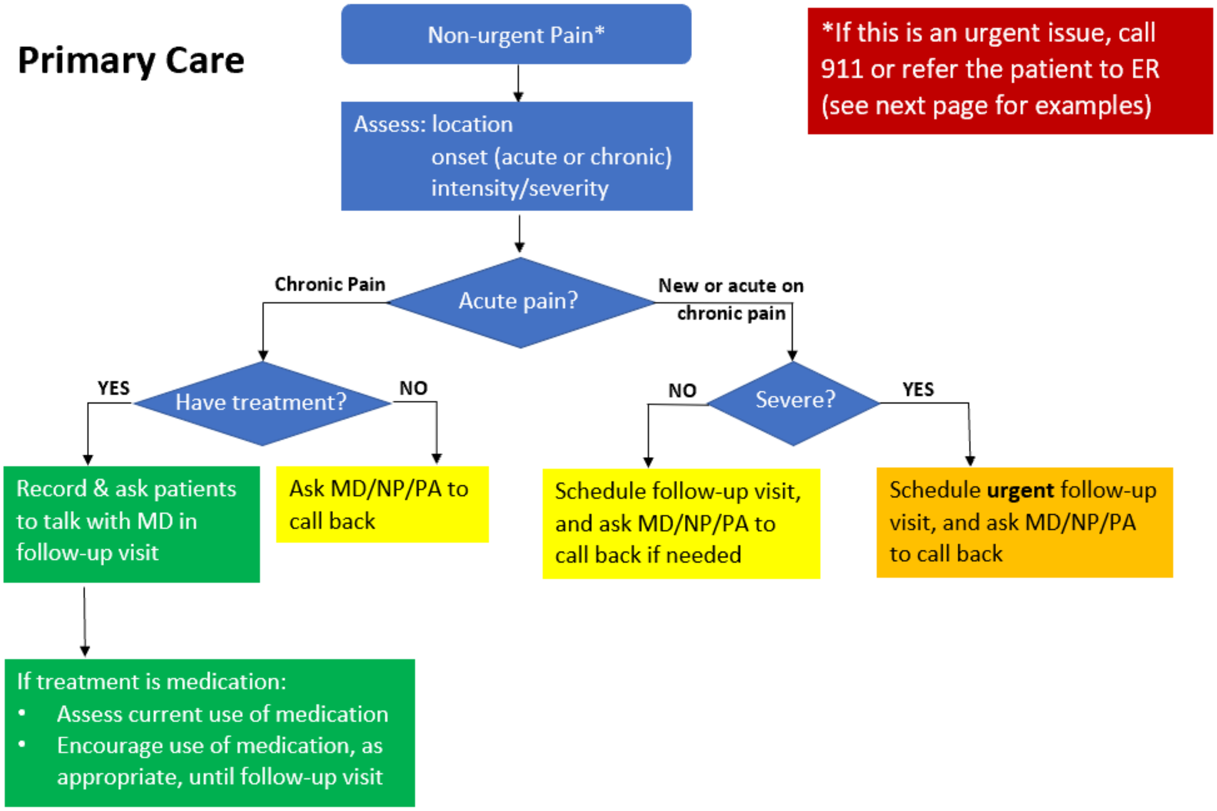

Cardiology

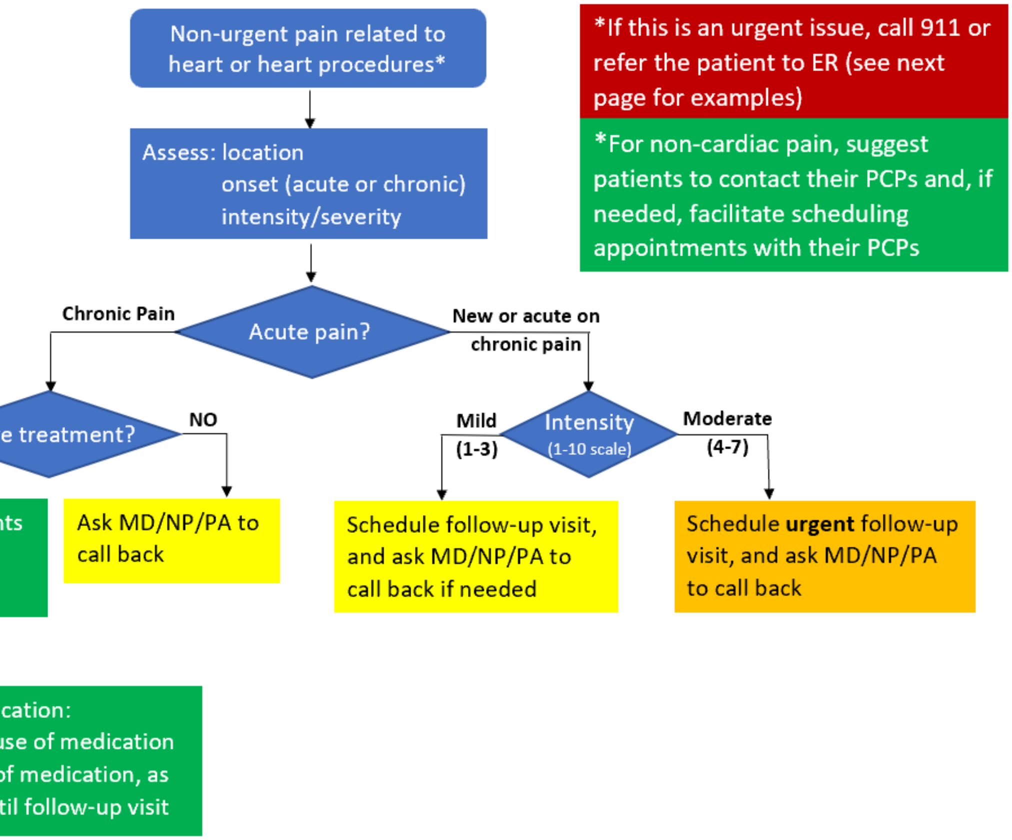

Examples of urgent pain:

- Chest pain similar to the pain related to patient's prior hospitalization
- Severe chest pain (7+ in 1-10 scale)
- Acute pain related to open wound fractures
- Acute pain related to changes in hemodynamic stability (e.g., low or high blood pressure or heart rate, change in O2 level, etc.)

Notes on Protocol:

1. Each clinic can add their own typical urgent cases.
2. The nurses are recommended to refer to the existing reference manuals (e.g., telephone triage protocols for nurses[1]) for urgent cases for common pain conditions (e.g., chest pain, back pain, and abdominal pain).
3. Clinics may wish to provide a resource link with contact information for pain management providers.

---

[1]Briggs JK. Telephone triage protocols for nurses. Lippincott Williams & Wilkins; 2007.
